# Primary Headache Disorders in Pediatric Irritable Bowel Syndrome: Characterizing IBS-Associated Migraine Highlights

**DOI:** 10.3390/children13091230

**Published:** 2026-09-11

**Authors:** Özben Akıncı Göktaş, Suna Selbuz

**Affiliations:** 1 Department of Pediatric Neurology, Ankara University School of Medicine, Balkiraz Mahallesi, Tıp Fakültesi Caddesi No: 1/4, Mamak, 06620 Ankara, Türkiye; 2 Department of Pediatric Gastroenterology, Ataturk Sanatoryum Training and Research Hospital, Sanatoryum Caddesi, Pınarbaşı Mahallesi, Ardahan Sokak No: 25, Keçiören, 06290 Ankara, Türkiye; kaymaksuna@gmail.com

**Keywords:** pediatric, irritable bowel syndrome, migraine, primary headache disorders, gut–brain axis

## Abstract

**Highlights:**

**What are the main findings?**

- Primary headache disorders affected more than half of children with IBS, with migraine and tension-type headache occurring at similar frequencies.
- Migraine in children with IBS was associated with a markedly higher prevalence of nausea, representing a clinically relevant, hypothesis-generating observation rather than evidence of a distinct migraine phenotype.
- Female sex, increasing age, and a positive family history of headache independently predicted migraine in pediatric IBS.

**What are the implications of the main findings?**

- Pediatric gastroenterologists should actively screen for headache disorders in children with IBS.
- The findings reinforce the role of gut–brain axis dysfunction in shaping migraine manifestations during childhood.
- Recognition of the higher prevalence of nausea among children with IBS and migraine may facilitate multidisciplinary clinical assessment and provide a basis for future prospective studies.

**Abstract:**

**Background/Objectives:** This study aimed to determine the prevalence of primary headache disorders in children and adolescents with irritable bowel syndrome (IBS), compare the clinical characteristics of IBS-associated and non-IBS headache populations, and identify factors associated with migraine and tension-type headache (TTH) in pediatric IBS. **Methods:** This retrospective observational study included 100 patients aged 6–18 years diagnosed with IBS according to the Rome IV criteria. All patients underwent systematic headache screening and neurological evaluation using the International Classification of Headache Disorders, 3rd edition. Age- and sex-frequency-matched control groups comprising 30 patients with migraine and 31 with TTH without gastrointestinal disease were included for comparison. Multivariable logistic regression analysis was performed to identify factors independently associated with migraine and TTH. **Results:** Primary headache disorders were identified in 61% of patients, including migraine in 30% and TTH in 31%. Migraine was significantly associated with female sex, increasing age, and a family history of headache. Compared with patients with migraine without IBS, those with IBS-associated migraine had a significantly higher prevalence of nausea (93.3% vs. 60.0%, *p* = 0.002), whereas headache frequency, duration, severity, photophobia, phonophobia, and vomiting were similar. No significant clinical differences were observed between IBS-associated and non-IBS TTH. **Conclusions:** Primary headache disorders, particularly migraine, are highly prevalent in pediatric IBS. The markedly increased frequency of nausea without differences in other headache characteristics represents a hypothesis-generating observation and may reflect an interaction between migraine-associated symptoms and the underlying gastrointestinal susceptibility associated with IBS, rather than evidence of a distinct migraine phenotype. Routine neurological assessment should be considered in children with IBS, particularly in female adolescents and those with a family history of headache.

## 1. Introduction

Irritable bowel syndrome (IBS) is one of the most common disorders of gut–brain interaction in children and adolescents. It is characterized by recurrent abdominal pain associated with altered bowel habits in the absence of structural abnormalities and is diagnosed according to the Rome IV criteria [1]. Increasing evidence suggests that IBS results from bidirectional dysfunction of the gut–brain axis involving alterations in the gut microbiota, visceral hypersensitivity, immune activation, and serotonergic signaling [2,3,4].

Migraine has been consistently associated with IBS, with studies demonstrating a higher prevalence of migraine in patients with IBS and a greater frequency of IBS among individuals with migraine [5,6,7,8]. These disorders share several clinical and pathophysiological features, including female predominance, chronic recurrent symptoms, gut–brain axis dysfunction, central and visceral hypersensitivity, low-grade inflammation, genetic susceptibility, and altered serotonin signaling [2,3,4,8,9]. In contrast, the association between IBS and tension-type headache (TTH) remains less well established, with inconsistent findings across studies [7,10,11,12].

Despite growing evidence linking IBS and primary headaches, pediatric data remain limited, particularly regarding TTH, headache characteristics, and factors associated with specific headache subtypes. Therefore, this study aimed to determine the prevalence of migraine and TTH in children and adolescents with IBS, compare headache characteristics between IBS-associated and non-IBS headache populations, and identify factors associated with headache subtypes in pediatric IBS.

## 2. Methods

### 2.1. Study Design and Population

**Figure 1:**
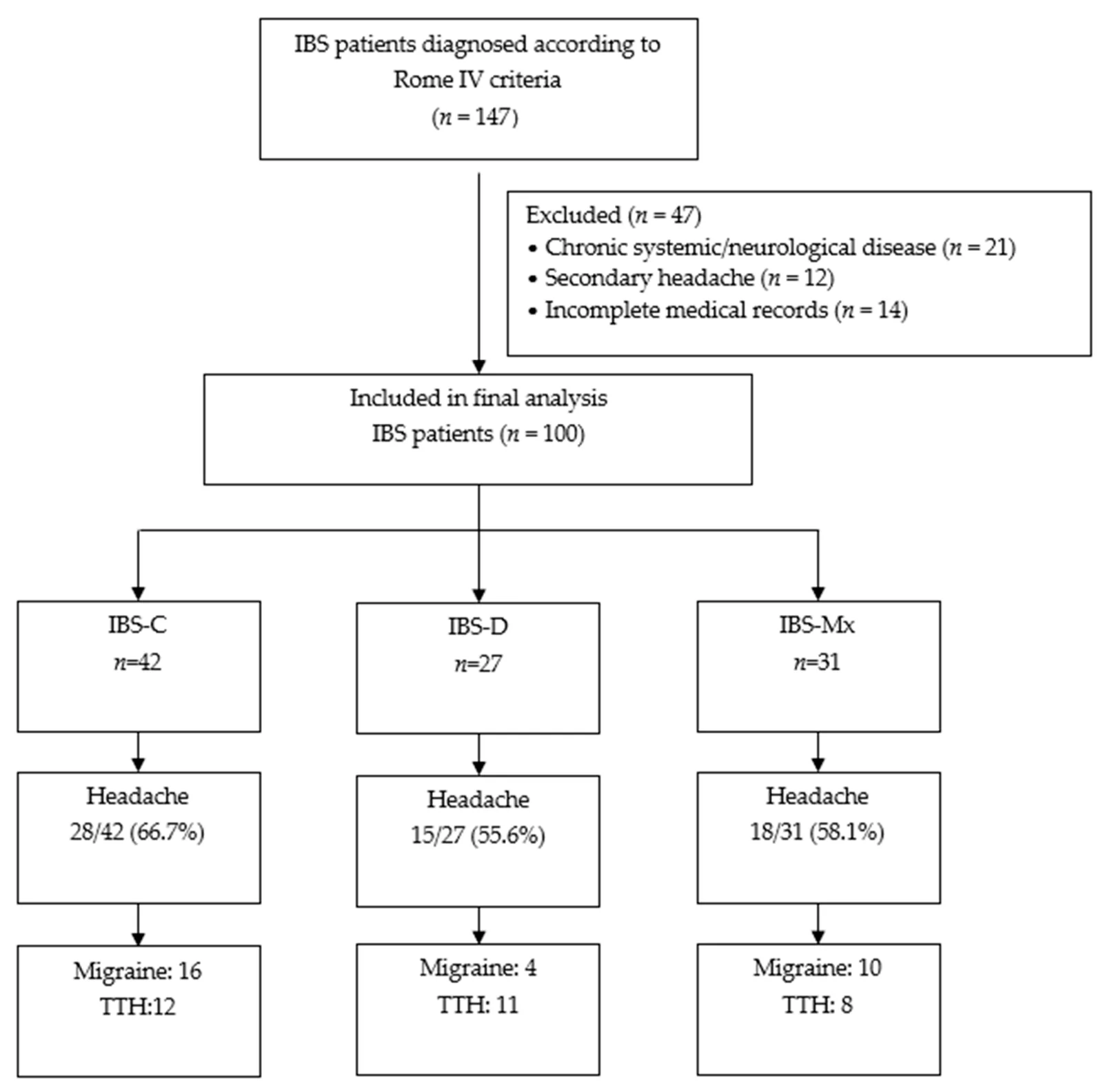
Flowchart of patient selection and distribution according to IBS subtype. IBS, irritable bowel syndrome; IBS-C, constipation-predominant IBS; IBS-D, diarrhea-predominant IBS; IBS-Mx, mixed-type IBS; TTH, tension-type headache.

This retrospective observational study was conducted at a tertiary care hospital using electronic medical records from 1 June 2021 to 30 April 2024. A total of 147 patients aged 6–18 years diagnosed with IBS according to the Rome IV criteria by the same pediatric gastroenterologist were screened. After excluding patients with psychiatric disorders, chronic systemic or neurological diseases (*n* = 21), secondary headache disorders (*n* = 12), or incomplete records (*n* = 14), 100 patients were included in the final analysis (Figure 1).

The study complied with the Declaration of Helsinki and was approved by the local institutional ethics committee.

### 2.2. IBS Classification and Headache Assessment

Patients were classified into three subtypes as constipation-predominant (IBS-C), diarrhea-predominant (IBS-D), or mixed-type (IBS-Mx) according to the Rome IV criteria [1]. No patients met the criteria for unclassified IBS (IBS-U).

All patients were systematically screened for headache using the same structured questionnaire developed by the study investigators, and the headache assessment was performed by the same pediatric neurologist. The questionnaire assessed the presence, duration, frequency, and clinical characteristics of headache, associated symptoms, and family history of headache. The duration and frequency of headaches were initially determined based on the patients’ and/or parents’ reports. Patients reporting a headache for at least three months underwent neurological evaluation by the same pediatric neurologist. A headache diary was provided to patients who reported headaches to document headache frequency and clinical characteristics during follow-up. Headache intensity was assessed using a 0–10 numerical rating scale based on the patient’s reported symptom intensity. Scores of 1–4 were classified as mild, 5–7 as moderate, and 8–10 as severe. Headache diagnoses were established according to the International Classification of Headache Disorders, 3rd edition (ICHD-3) [13]. Patients with secondary headaches were excluded. Primary headache disorders were classified as migraine or TTH; no other primary headache types were identified. Among the study population, 30 patients had migraine (IBS-Mig), 31 had TTH (IBS-T), and 39 had no headache.

### 2.3. Clinical and Laboratory Comparisons

Patients with and without headache were compared regarding age, sex, IBS subtype, hemoglobin, vitamin B12, and 25-hydroxyvitamin D (25-OHD) levels. These laboratory parameters were not specifically measured for the purposes of this study but had been obtained as part of routine clinical evaluation and were available in the medical records. They were compared to explore potential differences in commonly assessed clinical and nutritional parameters between patients with and without headache. Laboratory analyses included only patients who underwent routine clinical testing. IBS-C was additionally compared with the combined IBS-D/IBS-Mx group regarding headache prevalence and headache subtype distribution.

### 2.4. Control Group

Age- and sex-frequency-matched control groups consisting of 30 patients with migraine and 31 patients with TTH, without gastrointestinal or systemic disease, were retrospectively selected from the pediatric neurology outpatient clinic of the same institution. All headache diagnoses were established by the same pediatric neurologist. For the purposes of this study, “pure migraine” (PM) and “pure TTH” referred to patients diagnosed with migraine or TTH, respectively, without IBS or any other gastrointestinal or systemic comorbidity.

Patients with IBS-Mig were compared with those with pure migraine (PM) regarding length of headache history, attack duration, attack frequency (<10 days/month vs. ≥10 days/month), headache severity (mild, moderate, or severe), photophobia, phonophobia, nausea, and vomiting. Similarly, patients with IBS-associated TTH were compared with pure TTH regarding headache duration, attack duration, attack frequency, headache severity, photophobia, and phonophobia.

### 2.5. Statistical Analysis

All statistical analyses were performed using IBM SPSS Statistics for Windows, version 22.0 (Armonk, NY, USA: IBM Corp.). Normality was assessed using the Shapiro–Wilk test. Continuous variables are presented as mean ± SD or median (range), and categorical variables as frequencies and percentages. Comparisons were performed using Student’s *t*-test, Mann–Whitney U test, chi-square test, Fisher’s exact test, ANOVA, or Kruskal–Wallis test, as appropriate. A two-sided *p* < 0.05 was considered statistically significant. Separate multivariable binary logistic regression analyses were performed to identify factors independently associated with migraine and TTH among patients with IBS. Age, sex, and family history of headache were entered into the models, and results were expressed as odds ratios (ORs) with 95% confidence intervals (CIs). Given the relatively small number of migraine cases, the models were restricted to these prespecified clinically relevant variables to minimize the risk of overfitting.

## 3. Results

The mean age of the study population was 13.58 ± 3.67 years (range, 6–18 years), and 59 of the 100 patients were female. Primary headache disorders were identified in 61 patients, including 30 with migraine (IBS-Mig) and 31 with TTH (IBS-T). Patients with IBS-Mig were significantly older than those with IBS-T (15.28 ± 2.92 vs. 12.9 ± 3.6 years, *p* = 0.008) and were more frequently female (83.3% vs. 50.0%, *p* = 0.004). Forty-two patients had IBS-C, 27 had IBS-D, and 31 had IBS-Mx (Table 1).

No statistically significant differences were observed among IBS subtypes regarding age (*p* = 0.160), sex distribution (*p* = 0.396), headache prevalence (*p* = 0.602), or the distribution of migraine and TTH (*p* = 0.132) (Table 2).

### 3.1. Comparison of IBS-Mig and PM

When headache frequency was compared between the IBS-Mig and PM groups, 18 patients (60%) in the IBS-Mig group experienced attacks on <10 days per month, whereas 12 patients (40%) had attacks on ≥10 days per month. In the PM group, 13 patients (43.3%) had attacks on <10 days per month and 17 patients (56.7%) had attacks on ≥10 days per month. The difference between the groups was not statistically significant (*p* = 0.196) (Table 3).

In the IBS-Mig group, the median duration of headache history was 12 months (range, 3–72 months), and the median attack duration was 4.5 h (range, 2–72 h). In the PM group, the corresponding values were 13.5 months (range, 3–48 months) and 3 h (range, 2–72 h), respectively.

No statistically significant differences were observed between the groups (*p* = 0.961 and *p* = 0.662, respectively) (Table 3).

A family history of headache was present in 19 patients (63.3%) in the IBS-Mig group and in 18 patients (60.0%) in the PM group, with no statistically significant difference between the groups (*p* = 0.792).

With respect to headache severity, 26 patients (86.7%) in the IBS-Mig group described their pain as moderate and 4 patients (13.3%) as severe, whereas in the PM group, 22 patients (73.3%) reported moderate pain and 8 patients (26.7%) reported severe pain. The difference in headache severity between the groups was not statistically significant (*p* = 0.197) (Table 3).

Photophobia and phonophobia were reported in 25 (83.3%) and 28 (93.3%) patients in the IBS-Mig group, respectively, and in 28 (93.3%) and 24 (80.0%) patients in the PM group. The frequencies of both symptoms were comparable between the groups, with no statistically significant differences observed (*p* = 0.228 and *p* = 0.129, respectively) (Table 3).

Regarding associated gastrointestinal symptoms, nausea and vomiting were observed in 28 patients (93.3%) and 5 patients (16.7%) in the IBS-Mig group, respectively, compared with 18 patients (60.0%) and 4 patients (13.3%) in the PM group. Nausea was significantly more common in the IBS-Mig group than in the PM group, whereas no significant difference was observed between the groups with respect to vomiting (*p* = 0.002 and *p* = 0.718, respectively) (Table 3).

### 3.2. Comparison of IBS-T and PT

No statistically significant differences were observed between the IBS-T and PT groups regarding headache frequency (*p* = 0.529), headache history (*p* = 0.291), attack duration (*p* = 0.493), family history of headache (*p* = 0.316), headache severity (*p* = 0.224), photophobia (*p* = 0.195), or phonophobia (*p* = 0.553) (Table 4).

Similarly, headache localization and laterality were comparable between IBS-associated and non-IBS headache groups for both migraine (frontal localization, *p* = 0.213; bilateral distribution, *p* = 0.584) and TTH (frontal localization, *p* = 0.188; bilateral distribution, *p* = 0.246) (Table 3 and Table 4).

### 3.3. Comparison Between IBS-C and Other IBS Subtypes

Patients with IBS-C and the combined IBS-D/IBS-Mx group did not differ significantly regarding age (*p* = 0.089), sex (*p* = 0.613), headache prevalence (*p* = 0.327), migraine frequency (*p* = 0.134), TTH frequency (*p* = 0.652), photophobia (*p* = 0.356), phonophobia (*p* = 0.088), nausea (*p* = 0.133), or vomiting (*p* = 0.395).

### 3.4. Comparison of IBS Patients with and Without Headache

Among the 100 patients with IBS, 61 reported headache, and 39 did not. Patients with headache tended to be older (*p* = 0.068) and more frequently female (*p* = 0.090), although neither difference reached statistical significance (Table 5).

When laboratory parameters were compared, the mean hemoglobin (*p* = 0.691), vitamin B12 (*p* = 0.263), and 25-OHD levels (*p* = 0.594) were similar between patients with and without headache. Likewise, the family history of IBS did not differ significantly (*p* = 0.760). In contrast, a family history of headache was significantly more common among patients with headache than among those without headache (60.7% vs. 23.1%, *p* = 0.001) (Table 5).

In multivariable binary logistic regression analysis, female sex (odds ratio [OR] = 5.29, 95% confidence interval [CI]: 1.82–15.41, *p* = 0.002), increasing age (OR = 1.24, 95% CI: 1.07–1.44, *p* = 0.004), and a family history of headache (OR = 2.75, 95% CI: 1.14–6.66, *p* = 0.025) were independently associated with migraine in patients with IBS. No significant predictors were identified for TTH (sex, *p* = 0.213; age, *p* = 0.432; family history of headache, *p* = 0.161) (Table 6).

## 4. Discussion

Previous studies have consistently demonstrated a close association between IBS and migraine. A large pediatric study involving approximately 2000 children with migraine reported that IBS was associated with an almost fourfold increased risk of migraine [5], while the prevalence of IBS among migraineurs has been estimated to range from 4% to 40% [2]. Adult studies have similarly shown that 25–50% of individuals with IBS experience migraine or recurrent headache, compared with only 4–19% of healthy controls [14].

Consistent with these observations, migraine was identified in 30% of our IBS cohort, while primary headache disorders were present in 61%. This relatively high prevalence should be interpreted in the context of our tertiary-care setting. Patients referred to a tertiary center may have more persistent, complex, or clinically significant symptoms and may therefore be more likely to undergo comprehensive evaluation for both gastrointestinal and neurological complaints. Accordingly, the 61% prevalence observed in our study should not be directly extrapolated to the general pediatric population but rather interpreted as the proportion of primary headache disorders observed among children and adolescents with IBS evaluated in a tertiary-care setting. Referral and selection bias may have contributed to the relatively high prevalence observed in our cohort. The observed rates are substantially higher than those reported in a population-based pediatric study from Türkiye, where migraine and primary headache disorders were observed in 7.2% and 21% of healthy children, respectively [15]. However, these populations and study settings are not directly comparable, and the difference should therefore be interpreted cautiously. In pediatric migraine cohorts from Türkiye, nausea has been reported in 58.3–69.4% of patients, whereas vomiting has been observed in 27.7–31.2%; photophobia and phonophobia have been reported in 67–68% and 71.5–96.2%, respectively [16,17]. In contrast, our IBS-Mig group demonstrated a markedly higher prevalence of nausea (93.3%), while vomiting was less frequent (16.7%), despite similarly high rates of photophobia (83.3%) and phonophobia (93.3%). Moreover, nausea was significantly more common than in both our non-IBS migraine controls and previously published pediatric migraine cohorts.

Studies specifically investigating the association between IBS and TTH are limited, with the existing literature largely focusing on migraine. Martami et al. found that IBS was significantly more prevalent among adults with migraine but not among those with TTH [10]. Similarly, Le Gal et al. reported no significant association between functional gastrointestinal disorders and TTH in children [7]. In contrast, Park et al. observed a higher prevalence of probable TTH in children with functional constipation [11], suggesting that different disorders of gut–brain interaction may show distinct relationships with headache phenotypes. In our cohort, TTH was present in 31% of patients, exceeding prevalences reported in European (25.6%) [18], pooled pediatric (17%) [19], and Turkish (12.9–26.1%) populations [17,20]. The higher frequency of TTH observed in our IBS cohort highlights the importance of screening for headache disorders in pediatric IBS patients and may reflect shared pathophysiological mechanisms between functional gastrointestinal disorders and primary headache syndromes.

Consistent with previous findings in adults [12], no statistically significant differences were observed between IBS-C and other IBS subgroups regarding photophobia, phonophobia, nausea, vomiting, or the distribution of migraine and TTH.

Although nausea is not included in the formal diagnostic criteria for IBS, it commonly accompanies IBS and other functional gastrointestinal disorders [21]. Nevertheless, because IBS itself is associated with gastrointestinal symptom burden and altered gut–brain interactions, we cannot exclude the possibility that the presence of IBS contributed to the greater prominence or reporting of nausea during headache episodes. Thus, although nausea was assessed in the context of headache, the observed difference cannot be interpreted as evidence of a distinct migraine-specific phenotype. Rather, it may reflect an interaction between migraine-associated nausea and the underlying gastrointestinal susceptibility associated with IBS. The higher prevalence of nausea without a corresponding increase in vomiting observed in our IBS-Mig group may be related to altered gut–brain axis function. Previously proposed mechanisms, including dysregulated serotonergic signaling, enhanced vagal afferent sensitivity, and visceral hypersensitivity, may represent potential explanations for the preferential amplification of nausea perception rather than central emetic pathways [3,4,9]. However, these mechanisms were not directly assessed in the present study and should therefore be considered hypotheses rather than demonstrated mechanisms. Importantly, no significant differences were observed between the IBS-Mig and non-IBS migraine groups in other migraine characteristics, including attack frequency, duration, severity, photophobia, phonophobia, vomiting, or headache localization. Thus, the higher prevalence of nausea represents an isolated clinical observation rather than evidence that IBS alters the overall clinical characteristics of migraine. Prospective studies are needed to determine whether this observation reflects altered gut–brain interactions, enhanced visceral or sensory processing, or other mechanisms linking IBS and migraine.

Within our IBS cohort, female sex, increasing age, and a positive family history of headache were independently associated with migraine, whereas no significant predictors were identified for TTH. These findings are consistent with previous studies demonstrating that migraine and IBS share demographic characteristics and common gut–brain axis mechanisms [2,22]. These findings also support the hypothesis that TTH may have distinct or less overlapping pathophysiological drivers compared with migraine in the context of IBS. From a clinical perspective, these findings may help identify pediatric IBS patients who warrant closer neurological evaluation. Female adolescents with IBS and a positive family history of headache appear to constitute a subgroup at particularly increased risk of migraine, suggesting that targeted headache screening in this population may facilitate earlier diagnosis and management.

Several mechanisms have been proposed to explain the relationship between IBS and migraine, including gut–brain axis dysfunction, gut microbiota alterations, low-grade inflammation, food intolerances, genetic susceptibility, and impaired serotonergic signaling [2,8]. Supporting these mechanisms, dietary modification and microbiota-targeted therapies have been investigated as potential approaches for symptom management in both disorders [23,24]. However, the present study did not evaluate treatment response, and therefore our findings cannot establish whether IBS-directed interventions such as dietary modification or probiotic supplementation reduce migraine burden or improve quality of life. Thus, our findings are consistent with a possible clinical overlap between IBS and migraine; however, the underlying pathophysiological mechanisms were not directly assessed, and no causal relationship can be inferred from the present data. Altered gut–brain communication, serotonergic signaling, and visceral afferent processing have been proposed as potential mechanisms that could contribute to the greater prominence of gastrointestinal symptoms during migraine attacks. These mechanisms may provide a possible explanation for the higher prevalence of nausea observed in our IBS-Mig group, but this interpretation remains speculative and requires confirmation in prospective and mechanistic studies.

To our knowledge, this is one of the few pediatric studies simultaneously evaluating migraine and TTH in a well-characterized IBS cohort using standardized neurological assessment and comparison with non-IBS headache controls. The inclusion of multivariable regression analysis further enabled identification of factors independently associated with migraine, increasing the clinical relevance of our findings and providing a foundation for future prospective studies investigating gut–brain interactions in childhood.

The main limitations of this study are its retrospective cross-sectional design, single-center setting, and relatively small subgroup sample sizes, which may limit causal inference, generalizability, and statistical power. The tertiary-care setting may also have introduced referral and selection bias, potentially contributing to the relatively high prevalence of primary headache disorders observed in our cohort. Additionally, although nausea was assessed as a headache-associated symptom, the retrospective design limits our ability to determine the extent to which the underlying gastrointestinal symptom burden of IBS contributed to the reported nausea during headache episodes. Nevertheless, the study provides clinically relevant evidence regarding the association between IBS and primary headache disorders in children and adolescents and may serve as a basis for future prospective multicenter studies.

In conclusion, primary headache disorders, particularly migraine, are highly prevalent among children and adolescents with IBS. Migraine-associated nausea was significantly more frequent in children with IBS than in non-IBS migraine controls, whereas other migraine characteristics were comparable between groups. This isolated finding should be considered hypothesis-generating and may reflect an interaction between migraine-associated symptoms and the underlying gastrointestinal susceptibility associated with IBS rather than a distinct migraine phenotype. Female sex, increasing age, and a family history of headache independently identified children at greater risk of migraine within the IBS population. These findings emphasize the importance of incorporating neurological assessment into the routine evaluation of pediatric IBS and support further prospective studies investigating the clinical relationship between IBS and migraine and the potential role of gut–brain interactions.

## Figures and Tables

**Table 1:** Demographic and clinical characteristics of the IBS cohort.

Variable	Overall IBS Cohort (*n* = 100)
Age, years	13.58 ± 3.67 (range 6–18)
Sex, female/male	59/41
IBS subtype	IBS-C = 42; IBS-D = 27; IBS-Mx = 31
Headache present	61 (61%)
Headache subtype among patients with IBS (*n* = 61)	IBS-Mig = 30; IBS-T = 31
Age of headache groups, years	IBS-Mig: 15.28 ± 2.92 (range 8.2–18); IBS-T: 12.90 ± 3.60 (range 6.0–17.3) ***p* = 0.008**
Sex distribution of headache groups (female/male)	IBS-Mig: 25/5; IBS-T: 15/16 ***p* = 0.004**

IBS: irritable bowel syndrome; IBS-C: constipation-predominant IBS; IBS-D: diarrhea-predominant IBS; IBS-Mx: mixed-type IBS. IBS-Mig: irritable bowel syndrome-associated migraine; IBS-T: irritable bowel syndrome-associated tension-type headache. Data are presented as mean ± SD (range) or n (%).

**Table 2:** Comparison of clinical characteristics among IBS subtypes.

Variable	IBS-C (*n* = 42)	IBS-D (*n* = 27)	IBS-Mx (*n* = 31)	*p* Value
Age, years	12.85 ± 3.59	13.64 ± 3.74	14.53 ± 3.62	0.160
Female/Male	26/16	13/14	20/11	0.396
Patients with headache, n (%)	28 (66.7%)	15 (55.6%)	18 (58.1%)	0.602
Migraine, n (%) ^a^	16 (57.1%)	4 (26.7%)	10 (55.6%)	0.132
Tension-type headache, n (%) ^a^	12 (42.9%)	11 (73.3%)	8 (44.4%)	0.132

^a^ Percentages were calculated among patients with headache within each IBS subtype. Statistical significance was defined as *p* < 0.05.

**Table 3:** Comparison of demographic and headache characteristics between patients with IBS-associated migraine (IBS-Mig) and pure migraine (PM).

Variable	IBS-Mig (*n* = 30)	PM (*n* = 30)	*p* Value
Age	15.28 ± 2.92	15.13 ± 2.22	0.820
Sex, female/male	25/5	27/3	0.448
Headache frequency, n (%)			0.196
<10 days/month	18 (60)	13 (43.3)	
≥10 days/month	12 (40)	17 (56.7)	
Duration of headache history, months	12 (3–72)	13.5 (3–48)	0.961
Attack duration, hours	4.5 (2–72)	3 (2–72)	0.662
Family history of headache, n (%)	19 (63.3)	18 (60.0)	0.792
Headache severity, n (%)			0.197
Moderate	26 (86.7)	22 (73.3)	
Severe	4 (13.3)	8 (26.7)	
Photophobia, n (%)	25 (83.3)	28 (93.3)	0.228
Phonophobia, n (%)	28 (93.3)	24 (80.0)	0.129
Nausea, n (%)	28 (93.3)	18 (60.0)	0.002 *
Vomiting, n (%)	5 (16.7)	4 (13.3)	0.718
Frontal localization, n (%)	20 (66.7)	18 (60)	0.213
Bilateral headache, n (%)	19 (63.3)	21 (70)	0.584

* Data are presented as mean ± standard deviation, median (range), or n (%), as appropriate. Statistical significance was defined as *p* < 0.05.

**Table 4:** Comparison of demographic and headache characteristics between patients with IBS-associated tension-type headache (IBS-T) and pure tension-type headache (PT).

Variable	IBS-T (*n* = 31)	PT (*n* = 31)	*p* Value
Age	13 ± 3.60	13.16 ±2.97	0.840
Sex, female/male	15/16	15/16	1.000
Headache frequency, n (%)			0.529
<10 days/month	19 (61.3)	21 (67.7)	
≥10 days/month	12 (38.7)	10 (32.3)	
Duration of headache history, months	6 (3–60)	6 (1–60)	0.291
Attack duration, hours	1 (1–24)	1 (1–24)	0.493
Family history of headache, n (%)	18 (58.1)	14 (45.2)	0.316
Headache severity, n (%)			0.224
Mild	5 (16.1)	9 (29.0)	
Moderate	26 (83.9)	22 (71.0)	
Photophobia, n (%)	8 (25.8)	4 (12.9)	0.195
Phonophobia, n (%)	1 (3.2)	2 (6.5)	0.553
Frontal localization, n (%)	15 (48.4)	19 (61.3)	0.188
Bilateral headache, n (%)	21 (67.7)	25 (80.6)	0.246

Data are presented as mean ± SD, median (range), or n (%), as appropriate. Statistical significance was defined as *p* < 0.05.

**Table 5:** Comparison of IBS patients with and without headache.

Variable	IBS with Headache (*n* = 61)	IBS without Headache (*n* = 39)	*p* Value
Age, years	14.12 ± 3.45	12.75 ± 3.90	0.068
Female/Male	40/21	19/20	0.090
Hemoglobin, g/dL	13.98 ± 1.21 (*n* = 61)	14.07 ± 1.23 (*n* = 39)	0.691
Vitamin B12, pg/mL	355.0 ± 148.0 (*n* = 50)	393.3 ± 154.7 (*n* = 33)	0.263
25-Hydroxyvitamin D, ng/mL	14.63 ± 7.0 (*n* = 40)	15.57 ± 5.7 (*n* = 22)	0.594
Family history of IBS, n (%)	14 (23.0)	10 (25.6)	0.760
Family history of headache, n (%)	37 (60.7)	9 (23.1)	**0.001** *

* Data are presented as mean ± SD or n (%), as appropriate. The n values indicate the number of patients with available laboratory measurements for each parameter. Laboratory parameters were evaluated only in patients who underwent testing as part of routine clinical care. Abbreviations: IBS, irritable bowel syndrome. *p* < 0.05 was considered statistically significant.

**Table 6:** Multivariable binary logistic regression analysis of factors associated with migraine and tension-type headache (TTH) in patients with IBS.

Variable	Migraine (IBS Patients) OR (95% CI)	*p*-Value	TTH (IBS Patients) OR (95% CI)	*p*-Value
Female sex	5.29 (1.82–15.41)	**0.002**	1.72 (0.74–4.03)	0.213
Increasing Age (years)	1.24 (1.07–1.44)	**0.004**	0.96 (0.86–1.07)	0.432
Family history of headache	2.75 (1.14–6.66)	**0.025**	1.84 (0.79–4.29)	0.161

Abbreviations: OR, odds ratio; CI, confidence interval; TTH, tension-type headache. Note: Variables entered into the multivariable model included sex, age, and family history of headache. Odds ratios represent adjusted estimates from multivariable binary logistic regression.

## Data Availability

The data presented in this study are available from the corresponding author upon reasonable request.
